# JIA rehabilitation in 2014 and beyond: - a collaborative effort between child, family and health professional

**DOI:** 10.1186/1546-0096-12-S1-I26

**Published:** 2014-09-17

**Authors:** Janjaap Van Der Net

**Affiliations:** 1 Child Development and Exercise Center, Wilhelmina Childrens’Hospital, University Medical Center Utrecht, Utrecht, Netherlands

## 

With the introduction of biologics in pediatric rheumatology the options for effective treatment have increased considerably. Consequently the majority of children with JIA reach a remission in earlier stages of their disease and the impact on joint health, ambulation and functional ability of JIA have changed accordingly. This brings new perspectives for the (allied) health professional in the field of pediatric rheumatology. "Co-creation", family centered care, even family integrated care models are currently explored in this field. Multi-level; composed outcome measures (PRInTO-outcomes), patient rated outcome measures (PROM,s) are increasingly used in research and outcomes research, emphasis on family centered approaches deliver new measures such as the Family Needs Inventory What are the paradigm shifts we will face the coming decade, how does it influence our professional attitudes, skills and knowledge? What will be the added value of the (allied) healthprofessional in the coming decade?

## Disclosure of interest

None declared.

